# 

**Published:** 1910-12

**Authors:** 


### BOOKS RECEIVED

A Manual of Diseases of the Nose, Throat, and Ear. By E. Baldwin Gleason, M.D., Clinical Professor of Otology at the Medico-Chirurgical College, Philadelphia. Second revised edition. 12 mo. of 563 pages, profusely illustrated. Philadelphia and London: W. B. Saunders Company. 1910. (Flexible leather, $2.50, net.)

Diseases of the Stomach and Upper Alimentary Tract. By Anthony Bassler, M.D., Visiting Gastroenterologist to the People's Hospital, and Visiting Physician to St. Mark's Hospital Clinic, New York. Octavo, pp. 836. Illustrated. Philadelphia: F. A. Davis Co. 1910. (Cloth, $6.00.)

A Textbook of Bacteriology. By Philip Hanson Hiss, Jr., M.D., Professor of Bacteriology, College of Physicians and Surgeons, Columbia University, New York, and Hans Zinsser, M.D., Associate Professor in Charge of Bacteriology, Leland Stanford, Jr., University, Palo Alto, Cal. Octavo, pp. 745. Illustrated. New York and London: D. Appleton & Co. 1910. (Cloth, $3.75.)

Treatise on Diseases of the Skin. By Henry W. Stelwagon, M.D., Ph.D., Professor of Dermatology in Jefferson Medical College, Philadelphia. Sixth edition, thoroughly revised. Octavo, pp. 1195. Illustrated. Philadelphia and London: W. B. Saunders Co. 1910. (Cloth, $6.00, net.)

Bacteriology and Surgical Technic for Nurses. By Emily M. A. Stoney, Superintendent of Training School for Nurses, Rock Island, Ill. Third edition. 12 mo, pp. 311. Illustrated. Philadelphia and London: W. B. Saunders Co. 1910. (Cloth, $1.50.)

The Sexual Disabilities of Man and Their Treatment. By Arthur Cooper, Consulting Surgeon to the Westminster Dispensary, Formerly House Surgeon to the Male Lock Hospital, London. Second edition. 12 mo, pp. 204. Paul B. Hoeber, publisher, 69 East 59th Street, New York. (Cloth, $2.00.)

Diagnosis and Treatment of Diseases of Women. By Harry Sturgeon Crossen, M.D., Professor of Clinical Gynecology, Washington University, St. Louis. Second edition. Octavo, pp. 1025. With 744 engravings. St. Louis: C. V. Mosby Co. 1910. (Cloth, $6.00.)

Manual of Nursing. By Margaret Frances Donahoe, Formerly Superintendent of Nurses and Principal of Training School, Philadelphia General Hospital. 12 mo, pp. 489. Illustrated. New York and London: D. Appleton & Co. 1910. (Cloth, $2.00.)

Osteology and Syndesmology. By Howard A. Sutton, A.M., M.D., Assistant in the Department of Anatomy of the University of Pennsylvania, and Cecil K. Drinker, B.S.. 12 mo, pp. 225. Philadelphia: P. Blakiston's Son & Co. 1910. (Cloth, $1.50.)

State Board Examination Questions and Answers. Third edition. Reprinted from the Medical Record, New York: William Wood & Co. 1910. (Cloth, $3.00.)

The Practical Medicine Series. Ten volumes. Under the general editorial charge of Gustavus P. Head, M.D. Vol. VII. Pediatrics and Orthopedic Surgery, by Isaac A. Abt, M.D., and John Ridlon, M.D. Series 1910. Chicago: The Year Book Publishers. (Price, $1.25; entire series, $10.00.)

An Anatomical and Surgical Study of Fractures of the Lower end of the Humerus. By Astley Paston Cooper Ashurst, A.B., M.D., Prosector of Applied Anatomy in the University of Pennsylvania. The Samuel D. Gross Prize Essay of the Philadelphia Academy of Surgery, 1910. Octavo, pp. 163. Illustrated. Philadelphia and New York: Lea & Febiger. 1910. (Price, $2.75.)

Transactions of the sixteenth annual meeting of the American Laryngological, Rhinological and Otological Society, held in Washington, D. C., April 28, 29, and 30, 1910. Thomas J. Harris, M.D., Secretary.

